# The predictive factors of moral courage among hospital nurses

**DOI:** 10.1186/s13010-023-00141-9

**Published:** 2023-10-18

**Authors:** Hamideh Hakimi, Noushin Mousazadeh, Hamid Sharif-Nia, Roghieh Nazari, Maryam Dehghani

**Affiliations:** 1 Social Determinants of Health Research Center, Faculty of Nursing and Midwifery, Qazvin University of Medical Sciences, Qazvin, Iran; 2 Amol Faculty of Nursing and Midwifery, Mazandaran University of Medical Science, Sari, Iran; 3 Traditional and Complementary Medicine Research Center, Addiction Institute, Mazandaran University of Medical Sciences, Sari, Iran; 4 Instructor of Pediatric Nursing, Department of Nursing, Zeyinab (P.B.U.H) School of Nursing and Midwifery, Guilan University of Medical Sciences, Rasht, Iran

**Keywords:** Moral courage, Ethical climate, Nurses

## Abstract

**Background:**

Having moral courage is a crucial characteristic for nurses to handle ethical quandaries, stay true to their professional obligations towards patients, and uphold ethical principles. This concept can be influenced by various factors including personal, professional, organizational, and leadership considerations. The purpose of this study was to explore the predictors of moral courage among nurses working in hospitals.

**Methods:**

In 2018, an observational cross-sectional study was carried out on 267 nurses employed in six hospitals located in the northern region of Iran. The participants were selected through a simple random sampling technique. To collect data, a demographic information form was used along with two questionnaires. The first questionnaire was a standard survey on moral courage, while the second questionnaire was designed to assess the ethical climate. Linear regression was used to assess the predictors of moral courage.

**Results:**

Nurses had an average moral courage score of 87.07 ± 15.52 and an average moral climate score of 96.12 ± 17.17. The study showed that 16% of the variation in moral courage scores among nurses was explained by ethical climate and monthly overtime hours.

**Conclusion:**

This study underscores the significance of establishing an ethical work environment and minimizing overtime hours in order to enhance moral courage among nurses. These findings carry weight for both nursing practice and organizational policies focused on fostering ethical conduct within healthcare settings.

## Background

Nurses frequently face ethical dilemmas in their daily practice that challenge their moral convictions [1–3]. These situations may involve conflicting beliefs and values that can impact the quality of nursing care provided [4–7]. Possessing moral courage is essential to address these challenges, as it reinforces ethical conduct and enables nurses to carry out courageous actions in patient care [8, 9].

The notion of moral courage has been an integral aspect of nursing since the era of Florence Nightingale, with benevolence serving as a key principle of nursing practice [3]. This principle empowers individuals to make ethical decisions based on what is in the best interest of the patient [10].

Moral courage entails the ability to stand resolute and act ethically in the face of a dilemma, even if it means resisting external pressures to engage in unethical behavior [11]. Sekerka defines moral courage as the capacity to carry out ethical work and demonstrate benevolence despite external risks [12]. Individuals who possess moral courage make choices that align with what is right, even if it puts them at personal risk of losing their job, facing isolation from colleagues, or experiencing other negative outcomes. They stand firm for what is right, even if it means standing alone. Studies have shown that nurses need moral courage to effectively navigate ethical dilemmas, uphold their professional obligations to patients, and adhere to ethical codes of conduct [13]. Numminen et al. (2017) identified the elements of moral courage in nursing as true presence, moral integrity, responsibility, honesty, advocacy, commitment and perseverance, and personal risk. Moral sensitivity, conscience, and experience are prerequisites for moral courage which results in personal and professional growth and empowerment [14]. In a study on moral courage in nurses, it was defined as taking appropriate action, defending rights and implementing ethical principles to provide care to patients, even in the face of personal risks and threats [6].

Moral courage refers to the readiness to do what is right, uphold justice, and act in accordance with ethical principles when delivering healthcare services, even in the face of personal risks and threats [15]. This quality plays a crucial role in mitigating ethical distress [16, 17], fostering personal and professional growth [18, 19], and motivating individuals to acquire new skills and knowledge [12]. It is a personal characteristic that demands a steadfast commitment to ethical values, even when confronted with challenging circumstances. Without moral courage, the provision of nursing care can be compromised, leading to moral distress or unethical behavior [19].

Bandura's social cognitive theory (SCT) suggests that personal factors, behavior, and environment are interrelated and constantly influences each other [20]. Research has demonstrated that personal and professional factors [21], organizational culture, and leadership style [22] can influence the development of moral courage in nurses. The ethical climate of an organization also plays a crucial role in shaping moral courage [21, 23]. Advanced organizations often operate within complex moral environments that can significantly impact organizational performance. According to Hannah (2011) individuals must possess specific traits to enhance their behaviors when confronted with controversies [24].

In addition, technological advancements, shifts in therapeutic approaches and interventions, limited budgets, reduced hospitalization capacity, increased patient awareness of their rights, strengthened supervision systems, and health policies and regulations have brought about significant changes that underscore the importance of a better ethical climate [21]. Ethical climate aids individuals in identifying problems and serves as a framework for determining acceptable and unacceptable conduct [25].

As an aspect of organizational climate, ethical climate encompasses the ethical values and standards within the organization[26, 27]. For the first time, Victor and Cullen introduced the theory of moral climate. They classified ethical climate into five types: care, independence, law and code, rules, and instrumentality [28].

Olson defines ethical climate as the nurses' perceptions of ethical issues in their work environment, in other words, it is the individual's perceptions of the organization that affects the individual's attitude and behavior and becomes the framework of the nurse's behavior [29]. Ethical principles promote respect and honesty among staff, leading to increased job satisfaction and organizational achievement [25].

Furthermore, an ethical climate has been found to boost employee motivation, strengthen organizational commitment, and retain personnel [21].It also fosters a sense of ownership and reduces feelings of isolation among employees, ultimately contributing to organizational performance [30]. Conversely, an inadequate ethical climate can lead to understaffed departments, diminished motivation, and job dissatisfaction among nurses [21]. Studies conducted in Iran have revealed that nurses’ perception of the ethical climate in hospitals is at a moderate level [13]. However, few studies have examined the predictors of moral courage among nurses.. Therefore, the present study aimed to investigate the predictors of moral courage among nurses employed in hospitals.

## Methods

The present study was an observational cross-sectional study that was conducted in all educational treatment hospitals located in north of Iran from December 2018 to September 2019. The study population consisted of all clinical nurses working in these hospitals. The minimum sample size was calculated using G.Power (3.0.10) with a correlation coefficient between moral courage and ethical climate set at 10%,, α = 0.05, test power equal to 90%, effect size equal to 0.2, and a margin of error equal to 10%. Therefore, a sample size of 267 was obtained. A total of 267 nurses from six hospitals, 1480 clinical nurses, were selected using random sampling with the use of a random number table. Then, 45 nurses were selected from each hospital, whereby the researcher obtained the list of nursing staff from the hospital nursing management office and randomly selected the samples. Next, the work shift of participant was determined, and the questionnaires were given to the nurses during their work shift. At the end of the work shift, the questionnaires were collected, and if a questionnaire was incomplete, the researcher would collect it from the nurse during the next shift. Since ten questionnaires were incomplete, 7 nurses were randomly selected from these hospitals to complete the questionnaires. The Inclusion criteria for participants were holding at least an Associate degree or a Bachelor's degree in nursing, having one year of work experience, and being currently employed in one of the hospitals during the study period. The Exclusion criterion was failure to complete the questionnaires.

### Data collection tools

The study utilized three data collection instruments: a demographics form, the Professional Moral Courage Scale (PMC), and the Hospital Ethical Climate Survey. The PMC, developed by Sekerka et al. (2009), assesses moral courage across five dimensions: moral agency, multiple values, endures threat, goes beyond compliance, and moral goals. Each dimension contains three Likert-scale questions ranging from 1 (not at all) to 7 (always). The PMC has a minimum score of 15, a maximum score of 105, and a midpoint of 4 (sometimes). A higher score on this 15-statement scale indicates greater moral courage. The mean score of statements and total score of the tool represents the moral courage of respondent [12]. The Persian version of the PMC was evaluated for psychometric properties by Mohammadi et al. (2014) with a content validity index (CVI) structure of 81%. The Cronbach's alpha coefficient for the entire questionnaire was 0.85 [31].

The Hospital Ethical Climate Survey, developed by Olson et al. in 1998, comprises 26 items and measures five dimensions of ethical climate, including relationship with peers (4 items), patients (4 items), managers (6 items), hospital (6 items), and physicians (6 items). The items were scored using five-point Likert scale ranging from 1 (almost never true) to 5 (almost always true) [32]. A higher score on this survey indicates a more positive ethical climate. The Persian version of this survey was evaluated for reliability by Mobasher and et al. (2004), with a Cronbach alpha of 0.9 [33]. The Cronbach's alpha values for both instruments in this study were 0.91. Permission to use the instruments was obtained from their respective developers prior to the study.

### Statistical analysis

The statistical analysis was performed using SPSS (v.22). The Kolmogorov–Smirnov test was utilized to assess the normal distribution of the continuous quantitative data. Simple linear regression was applied to analyze the predictive variables of moral courage. The variables that were found to be significant in the simple linear regression were then subjected to multiple linear regression analysis. The consistency and independence among the remaining variables were assessed using the variance inflation factor and Durbin-Watson tests (*p* < 0.05). The level of missing data was determined using the “Multiple Pattern” command. The total response rate of the questionnaires was 97%.

## Results

The study found that the mean age of men, 31.56 (SD = 8.08, CI%95: 29.33 to 35.76), was lower than that of female nurses,34.45 (SD = 7.65; CI%95: 33.35 to 41.50). The demographics characteristics of the nurses are presented in Table 1.

**Table 1:** Demographics of the participants

**Demographics**		N(%)
**Gender**	Female	213 (79.8)
	Male	54 (20.2)
**Marital status**	Married	191(71.5)
	Single	76 (28.5)
**Education**	Associates’ degree	12(4.5)
	Bachelors’ degree	239 (89.5)
	Masters’ degree	16 (6.0)
**Participation in ethics congress**	Yes	91(30.3)
	No	187 (69.7)
**Satisfaction with salary**	Low	179 (67.0)
	Moderate	88 (33.0)
	High	0 (0)
**Satisfaction with manager**	Low	128 (47.9)
	Moderate	129 (48.3)
	High	10 (3.8)
**Employment status**	permanent	106(39.7)
	A five-year contractual	80(30.0)
	A two-year contractual	42(15.7)
	contractual	39(14.6)
	Age	33.86 (SD=7.86)
	Experience in the current ward	6.08 (SD=6.65)
	Nursing experience	9.90 (SD=6.65)
	Overtime work per month(hrs)	58.10 (SD=6.65)

The mean score of moral courage and ethical climate were 87.07 (SD = 15.52; CI%95: 85.22 to 88.96) and 96.12 (SD = 17.17; CI%95: 92.05 to 96.19), respectively. The results of the independent t-test revealed a significant relationship between gender, and moral courage (Table 2). Furthermore, the Pearson correlation test demonstrated a significant correlation between age, nursing experience, overtime hours, and ethical climate with moral courage (Table 3). However, the other Variables did not show a significant correlation with moral courage (Table 4).

**Table 2:** Comparison of Mean of Moral Courage with Demographic Variables (t-test)

Variables	Mean	SD	t	df	P
**Gender**					
Female	89.25	13.95	4.58	68.61	0.01
Male	78.75	18.75			
**Marital status**					
Married	88.2	14.4	2.07	264	0.07
Single	83.85	17.55			
**Participation in ethics congress**					
Yes	87.6	15.06	0.44	261	0.9
No	86.7	15.06	1.03		
**Satisfaction with salary**					
Low	86.25	16.35	1.03	259	0.32
Moderate	88.35	13.8

**Table 3:** Comparison of Mean of Moral Courage with Demographic Variables (Pearson)

Variables	r	P
**Age**	0.12	0.03
**Experience in the current ward**	-0.7	0.2
**Nursing experience**	0.12	0.05
**Overtime work per month (hrs)**	-0/19	0.002
Ethical climate	0.23	0.0001

**Table 4:** Comparison of Mean of Moral Courage with Demographic Variables (ANOVA)

Variables	Mean	SD	df	P	F
**Education**					
Associates’ degree	93.3	12.45	4	0.57	0.73
Bachelors’ degree	86.55	15.6			
Masters’ degree	88.2	16.65			
**Employment**					
permanent	84	19.05	4	0.1	1.94
A five-year contractual	90	10.35			
A two-year contractual	86.7	15.15			
One- year employment contract	88.95	13.35			
**Satisfaction with managers**					
Low	84.45	17.7	3	0.1	1.97
Moderate	88.8	13.65			
High	91.5	8.1			

However, the regression analysis indicated that only two variables, namely monthly overtime work hours and perception of ethical climate, remained in the model (Table 5).

**Table 5:** Predictors Factor of Moral Courage in Nurses

Regression index	**Not adjusted (simple)**			**Adjusted (multiple)**		
Variable	**B**	***P* Value**	**CI 95%**	**B**	***P* Value**	**CI 95%**
Age	0.254	0.039	0.013 to 496	-	-	-
Gender (female/male*)	-10.42	0.001	-14.92 to -50.90	-	-	-
Marital status (Single*/ Married)	-4.375	0.039	8.052 to 0.22	-	-	-
Overtime work per month (hrs)	0.081	0.002	0.13 to 0.04	0.06	0.001	1.13 to 0.018
Satisfaction with management (low, moderate, high* )	3.674	0.18	640 to 6.635	-	-	-
Ethical climate	0.213	0.001	1.09 to 0.319	0.183	0.001	0.83 to 0.283

*Reference

As shown in the table, these variables can predict moral courage. Overall, 16% of the variation in moral courage is explained by monthly overtime work hours and ethical climate.

## Discussion

This study aimed to identify predictive factors of moral courage among nurses and assess their level of moral courage. The findings showed that the moral courage of nurses was high, which is consistent with previous studies [34–38]. Some studies found a correlation between age, work experience, employment status, and participation in ethics classes with moral courage [35, 36]. while others found no significant relationship between these variables and moral courage [34].

Identifying the factors that predict moral courage is crucial for creating a suitable environment that promotes this vital human virtue in the nursing community. In this study, variables such as age, gender, marital status, overtime hours per month, satisfaction with managers, and ethical climate were found to be correlated with moral courage. However, only two variables, overtime hours per month and perception of ethical climate, were identified as predictors of moral courage. The findings suggest that improving the ethical climate in hospital departments can increase the moral courage of nurses.

Ethical climate has been shown to impact various organizational events, such as organizational commitment, job satisfaction, intention to change ward, moral stress, and organizational citizenship behavior [31, 39, 40]. It has also been found to have a direct relationship with individual performance in an organization [40]. Suhonen et al. (2011) have argued about the effects of ethical climate in an organization on moral courage [21]. Therefore, improving the ethical climate in hospitals may lead to a more favorable environment that enhances moral courage among nurses.

The healthcare system is currently undergoing rapid changes to meet the growing needs of society. These changes have led to an increase in the number and complexity of ethical questions that nurses face. A positive ethical climate that emphasizes moral values can provide nurses with the courage to question work processes and promote improvements in health services. Our findings are consistent with a previous study that found a positive ethical climate was associated with increased moral courage among nurses [31]. This suggests that ethical climate can be modified to improve healthcare and facilitate ethical decision-making.

Another finding of our study was that high work hours significantly increased moral courage among nurses. While, there is no specific study examining the relationship between work hours and moral courage in nurses, an analysis by Numminen (2017) on the concept of moral courage suggested that experience was a precondition for developing moral courage [14]. In contrast, other studies have found no significant relationship between work hours and moral courage [34, 35]. This finding can be explained by SCT, which highlights the reciprocal and continuous relationship between personal factors, behavior, and environment [20]. Longer work hours provide more opportunities for social and environmental interactions, which may facilitate changes in personal factors, such as moral courage among nurses.

Regression analysis revealed that while ethical climate and work hours were predictors of moral courage among nurses, they only accounted for 16% of the variance. Therefore, future research should identify other key predictors of moral courage among nurses. While overtime work hours and ethical climate had some influence on moral courage, their contribution was not significant. Hence, it is recommended that organizations focus on improving the adjustable variable of ethical climate to enhance moral courage in nurses. Further research is needed to explore other factors that may impact moral courage in nurses.

## Conclusions

Moral courage is a crucial concept in the nursing profession, as it plays a significant role in providing patient care and decision-making. Therefore, promoting moral courage is essential. Our study found that overtime work hours and ethical climate in the work environment were factors that affected this concept. Other organizational characteristics should also be investigated to determine their relationship with moral courage. By prioritizing moral courage as a factor that can improve nursing care quality, organizations can enhance their success and survival.

It is important to note that the promotion of moral courage among nurses should not be limited to individual efforts. Instead, organizations must create a supportive work environment that encourages and rewards ethical behavior. This can be achieved through the development of policies and procedures that promote ethical behavior, training programs that focus on ethical decision-making, and the creation of a culture that fosters open communication and transparency.

In conclusion, our study emphasizes the importance of promoting moral courage among nurses. By identifying factors that affect this concept, such as overtime work hours and ethical climate in the work environment, organizations can take steps to enhance their success and survival. Furthermore, promoting moral courage should not be limited to individual efforts but should be a collective effort that involves the entire healthcare organization.

## Data Availability

All data generated or analyzed during this study are included in this published article.

## Abbreviations

SCT: Social cognitive theory
PMC: Professional Moral Courage
SPSS: Statistical Package for Social Science
